# Intracoronary Imaging in the Detection of Vulnerable Plaques

**DOI:** 10.1007/s11886-016-0705-1

**Published:** 2016-02-15

**Authors:** Jonathan A. Batty, Shristy Subba, Peter Luke, Li Wing Chi Gigi, Hannah Sinclair, Vijay Kunadian

**Affiliations:** Institute of Cellular Medicine, Newcastle University, 3rd Floor, William Leech Building, Newcastle Upon Tyne, NE2 4HH UK; Freeman Hospital, Newcastle Upon Tyne NHS Foundation Trust, Newcastle Upon Tyne, NE7 7DN UK; Chinese University of Hong Kong, Hong Kong SAR, People’s Republic of China

**Keywords:** Interventional cardiology, Imaging, Coronary artery disease, Intravascular ultrasonography, Near-infrared spectroscopy, Optical coherence tomography

## Abstract

Coronary artery disease is the result of atherosclerotic changes to the coronary arterial wall, comprising endothelial dysfunction, vascular inflammation and deposition of lipid-rich macrophage foam cells. Certain high-risk atherosclerotic plaques are vulnerable to disruption, leading to rupture, thrombosis and the clinical sequelae of acute coronary syndrome. Though recognised as the gold standard for evaluating the presence, distribution and severity of atherosclerotic lesions, invasive coronary angiography is incapable of identifying non-stenotic, vulnerable plaques that are responsible for adverse cardiovascular events. The recognition of such limitations has impelled the development of intracoronary imaging technologies, including intravascular ultrasound, optical coherence tomography and near-infrared spectroscopy, which enable the detailed evaluation of the coronary wall and atherosclerotic plaques in clinical practice. This review discusses the present status of invasive imaging technologies; summarises up-to-date, evidence-based clinical guidelines; and addresses questions that remain unanswered with regard to the future of intracoronary plaque imaging.

## Introduction

Despite significant advancements in pharmacological and interventional management, coronary artery disease (CAD) remains the leading cause of morbidity and mortality worldwide [1, 2]. The first clinical manifestation of CAD is often acute coronary syndrome (ACS), comprising ST-elevation and non-ST-elevation acute myocardial infarction (MI) and unstable angina pectoris. CAD comprises a continuum of pathophysiological changes to the intima and media of the coronary arterial wall, characterised by endothelial dysfunction; vascular inflammation; accumulation of lipid, calcium and cellular debris; and in certain plaques, coronary artery stenosis. Long-established as the gold standard to evaluate the presence, location and extent of stenosis associated with CAD, invasive coronary angiography provides a two-dimensional representation of the coronary lumen but is incapable of visualising the composition of the atherosclerotic plaque [3]. Approximately two thirds of acute coronary events occur due to the rupture of lesions with non-critical (≤50 %) stenosis on angiography and are characterised by specific histological features conferring vulnerability [4–6].

Impelled by the inherent limitations of coronary angiography, complementary imaging modalities have emerged, capable of visualising the components of the arterial wall and characterising coronary plaques. Intravascular ultrasound (IVUS), optical coherence tomography (OCT) and near-infrared spectroscopy (NIRS) are techniques capable of robust, qualitative and quantitative evaluation of the coronary artery wall, and plaques contained within [7–9]. Emerging clinical evidence suggests that intracoronary imaging is a safe and effective adjunct to angiography, both as a research tool and in clinical practice, providing superior diagnostic precision regarding plaque architecture, composition and severity and enabling incremental risk stratification. However, despite accumulating evidence, guidelines regarding the use of intracoronary imaging for the detection of vulnerable coronary plaques remain limited [10, 11].

The purpose of this review is to provide a critical overview of the applications of in vivo intracoronary imaging techniques, in the identification of high-risk, vulnerable atherosclerotic plaques. This review will discuss the available imaging technologies, consider their relative benefits and limitations and explore how the invasive imaging of high-risk, vulnerable plaques may advance in the future.

## The Vulnerable Atherosclerotic Plaque

The concept of the high-risk or *vulnerable* plaque emerged from landmark, longitudinal studies of patients with ST-elevation MI. Analysis of serial and pre- and post-infarct angiography demonstrated contrary to popular belief that most infarctions were not caused by severely stenotic lesions [5, 12]. The plaques attributed to causing infarction were moderately sized at baseline. This is explained by expansive remodelling in the arterial wall; the lumen remains uncompromised until the plaque achieves a critical volume. Thus, the degree of stenosis is not the primary determinant of plaque rupture.

A post-mortem, histopathological study examined the hearts of 113 men, 41 of which had thrombosis secondary to vulnerable plaque rupture. Of these, 95 % of lesions had thin fibrous caps, with macrophage infiltrate overlying a lipid-rich pool [6]. The thin-cap fibroatheroma (TCFA) represents a specific morphology of vulnerable plaque, characterised by a thin fibrous layer overlying a large core of lipid-rich necrotic debris and associated with expansive arterial remodelling [13, 14]. TCFA is most frequently observed in the proximal coronary vasculature. Key characteristics conferring vulnerability to rupture include the presence of (i) a thin fibrous cap (<65 μm); (ii) a large, lipid-enriched, necrotic core (>40 % total plaque volume); (iii) localised macrophage infiltration and inflammation; (iv) vascular remodelling; (v) densely calcified areas (>10 %); and (vi) large plaque volume [15]. Inflammation secondary to shoulder macrophage activation, endothelial denudation, superficial thrombocyte aggregation and haemodynamically significant stenosis (>90 %) further predispose to rupture and subsequent atherothrombosis [16].

The majority of vulnerable plaques remain clinically silent until the development of acute MI, emphasising the importance of early identification, enabling prognostic stratification and optimisation of management (e.g. risk factor modification; secondary preventative pharmacotherapy).

## IVUS

### IVUS Technology

Greyscale IVUS uses the amplitude of reflected ultrasound waves to generate an image [17–19]. Many successful applications of IVUS in research have greatly advanced the understanding of the pathophysiology and natural history of coronary atherosclerosis, such as providing the first in vivo evidence of expansive arterial remodelling [20]. IVUS permits detailed evaluation of the coronary artery lumen, wall and plaque area and enables both qualitative and quantitative and pre- and post-intervention assessment of lesion morphology, to a depth of 5–10 mm, with an axial spatial resolution of 100–200 μm at frequencies ranging from 20 to 45 MHz. The IVUS catheter is mounted to an automated pullback device, which withdraws the catheter at a pre-set speed (e.g. 0.5 mm s^−1^), enabling the acquisition of a cylinder, representative of a length of an artery.

**Fig. 1:**
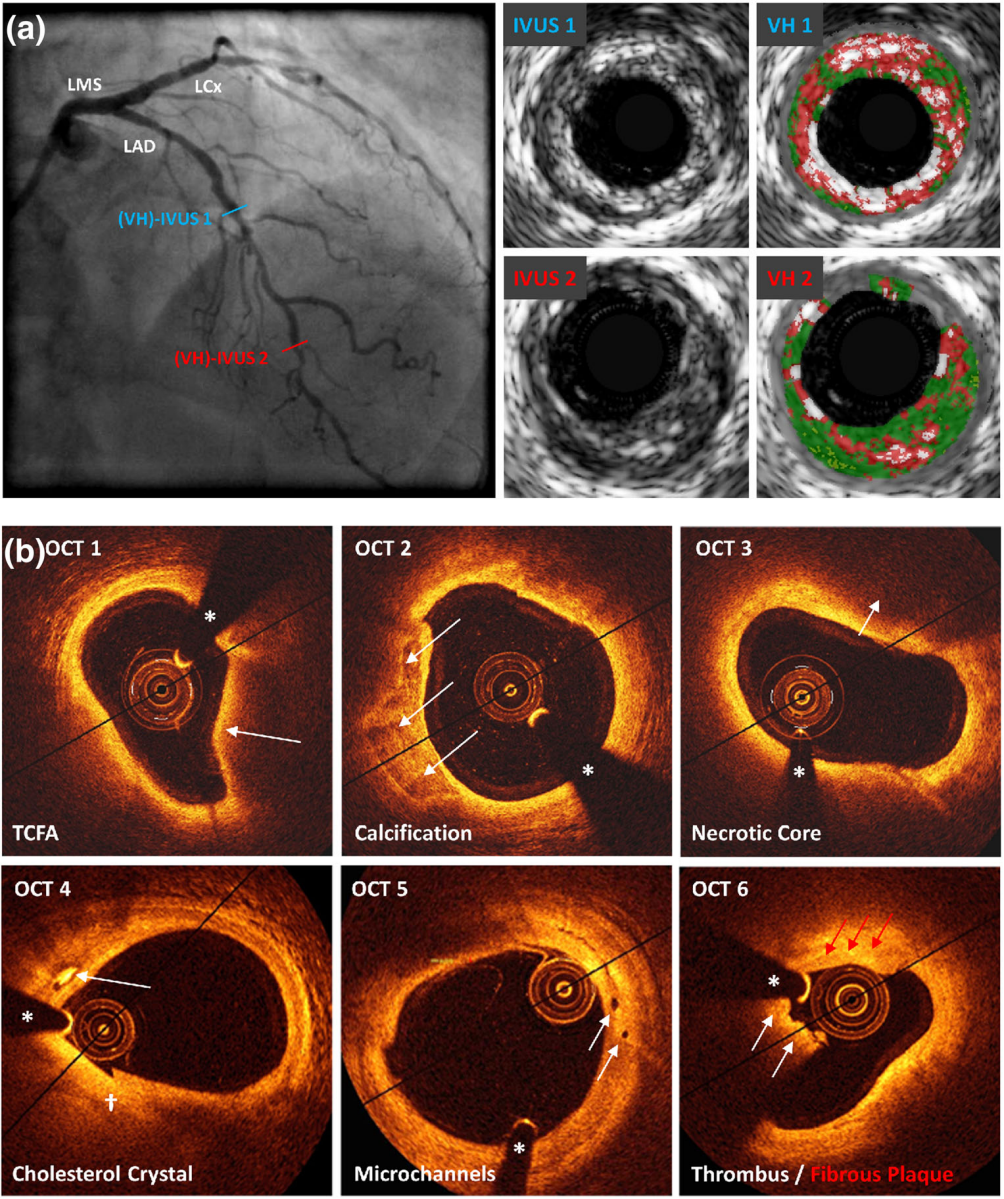
Examples of intracoronary imaging modalities. **a** (VH-)IVUS. The coronary angiogram shows the left anterior descending artery (RAO cranial), demonstrating minimal stenosis, but multiple vulnerable plaques are visualised on IVUS. Cross-sectional images demonstrate calcified TCFA (*blue line*, *IVUS1* and *VH1*) and non-calcified TCFA (*red line*, *IVUS2* and *VH2*). Keys to VH-IVUS: *dark green* fibrous tissue, *light green* fibro-fatty tissue, *red* necrotic core, *white* dense calcium. **b** OCT. Several examples of features associated with plaque vulnerability are presented, including: *OCT1* TCFA (*arrow* indicates thin fibrous cap), *OCT2* coronary arterial calcification (*arrows* indicate well-demarcated calcification), *OCT3* necrotic core (*arrows* indicate lipid pool/necrotic core), *OCT4* presence of cholesterol microcrystal (*arrow* indicates well-demarcated crystal structure), *OCT5* microchannels (*arrows* indicate two separate channels) associated with a non-obstructive lesion and *OCT6* (*white arrows* indicate low-attenuation white thrombus; *red arrows* indicate highly fibrous plaque). *Diag* diagonal artery, *IVUS* intravascular ultrasound, *LAD* left anterior descending artery, *LCx* left circumflex artery, *LMS* left main stem, *OCT* optical coherence tomography, *OM* obtuse marginal artery, *prob* probability, *RAO* right anterior oblique view, *TCFA* thin-cap fibroatheroma, *VH* virtual histology. *Asterisk* indicates guidewire artefact; *dagger* indicates seam line artefact

The highly reproducible nature of vessel and plaque measurements using IVUS has been robustly validated [21]. The greyscale image provided by IVUS permits classification of multiple tissues: (i) soft, with echogenicity less than nearby adventitia; (ii) calcified, with echogenicity greater than nearby adventitia; (iii) fibrous, with intermediate echogenicity; and (iv) mixed, with several visible acoustic signals [22, 23]. However, IVUS lacks sensitivity in the identification of lipid-rich plaque (approximately two thirds of all plaques) and has suboptimal spatial resolution to permit a detailed analysis of plaque characteristics, such as visualisation or measurement of fibrous cap thickness (Fig. 1a) [17, 24]. In addition, IVUS cannot reliably identify a thrombus [18]. High-frequency transducers (>40 MHz) yield increased resolution, with improved plaque characterisation, at the cost of greater artefact due to reflection of ultrasound from the blood and reduced depth of penetration. Although this may cause confusion in interpreting margins between structures (e.g. the lumen-tissue border), this may be resolved by catheter-guided saline injection [25].

### Integrated Backscatter IVUS and Virtual Histology IVUS Technology

Qualitative assessment of plaque morphology using greyscale IVUS requires significant post-processing; the development of plaque characterisation algorithms, such as integrated backscatter IVUS (IB-IVUS) and virtual histology IVUS (VH-IVUS), reduces operator-dependent image interpretation. These techniques augment greyscale IVUS images with a colour map of the plaque architecture, stratified by a tissue type. IB-IVUS uses time domain information extracted directly from the radio-frequency signal to characterise plaque constituents, with high sensitivity and specificity [26, 27]. VH-IVUS is performed using either a 20-MHz phased array or a 45-MHz rotational catheter and uses spectral analysis of the frequency and amplitude of ultrasound signals reflected by tissues to characterise plaque components, using an algorithm derived from known tissue types, to detect fibrous tissue (FT, dark green), fibro-fatty tissue (FFT, light green), necrotic core (NC, red) and dense calcium (DC, white; Fig. 1a) [28–31].

The sensitivity and specificity of VH-IVUS is broadly similar to that of IB-IVUS; the degree of concordance between VH-IVUS and in vitro histopathology (following atherectomy) was 87.1–96.5 %, dependent on tissue [32–34]. However, discrepant results exist with regard to the identification of NC; several studies demonstrate poor associations between VH-IVUS and histological analysis [35, 36]. This is partly due to the fact that the initial VH-IVUS spectral classification did not differentiate between necrotic and calcified areas, combining the two into *calcified-necrotic* regions [37].

## IVUS, IB-IVUS, VH-IVUS and Vulnerable Plaques

### Definitions

The definition of TCFA on IVUS should reflect the histopathological characteristics of TCFA but must respect the limitations of the imaging modality. As such, given the limited resolution of IVUS, the absence of visible fibrous tissue overlying a lipid core implies a cap thickness of ≤100–200 μm. As such, the majority of IVUS studies adopt a simplified definition of TCFA, as a focal necrotic core-rich lesion, without evidence overlying fibrous tissue [38].

### IVUS and ACS

The multi-centre, PROSPECT (Providing Regional Observations to Study Predictors of Events in the Coronary Tree) cohort study recruited *n* = 697 patients with ACS undergoing percutaneous coronary intervention (PCI) to undergo three-vessel greyscale and VH-IVUS [39]. The primary endpoint was a composite of MI, rehospitalisation, cardiac arrest and cardiac-related mortality. A total of 596 TCFA cases were visualised in 313 patients. After a median of 3.4-year follow-up, the rate of the primary endpoint was 20.4 %; events during follow-up were attributed to the culprit lesion in only 12.9 %. Most of the non-culprit lesions causative of follow-up events were angiographically mild at recruitment (mean ± standard deviation diameter stenosis 32.3 ± 20.6 %). Multivariate analysis demonstrated that non-culprit lesions associated with events were more likely to be associated with (i) plaque burden of ≥70 % (hazard ratio (HR) 5.03, 95 % confidence interval (CI) 2.51–10.11; *p* < 0.001), (ii) minimal luminal area of ≤4.0 mm^2^ (HR 3.21, 95 % CI 1.61–6.42; *p* = 0.001) or (iii) underlying IVUS-TCFA (HR 3.35, 95 % CI 1.77–6.36; *p* < 0.001). However, 48 % of the lesions causing events during follow-up were not imaged at baseline in PROSPECT; IVUS imaging was limited to the proximal and middle portions of the arteries, where the vessel diameter was sufficient to accommodate the IVUS catheter. Crucially, this study suggests that adverse cardiac events have a propensity to occur, with reasonable frequency, at the site of non-culprit lesions.

The VIVA (VH-IVUS and Vulnerable Atherosclerosis) study also prospectively evaluated the association between TCFA and adverse coronary events in *n* = 170 patients, with stable angina and ACS, undergoing PCI [28]. All patients underwent a three-vessel VH-IVUS pre- and post-PCI. The primary endpoint consisted of myocardial infarction, unplanned revascularisation or death. At a median of 1.75-year follow-up, the primary endpoint was attributed to 19 lesions (6 culprit and 13 non-culprit) in 16 patients. The same non-culprit factors identified in PROSPECT also associated with the primary endpoint: plaque burden (HR 8.13, 95 % CI 1.63–40.56; *p* = 0.011), luminal area (HR 2.91, 95 % CI 1.07–7.91; *p* = 0.036) and VH-TCFA presence (HR 7.53, 95 % CI 1.12–50.55; *p* = 0.038). This study demonstrates the prognostic significance of IVUS in the identification of patients at high risk of experiencing adverse cardiovascular outcomes.

Recently, the ATHEROREMO-IVUS (European Collaborative Project on Inflammation and Vascular Wall Remodelling in Atherosclerosis-Intravascular Ultrasound) study assessed the value of IVUS in patients with CAD [40••]. A total of *n* = 581 patients undergoing PCI for stable angina (*n* = 263) and ACS (*n* = 318) underwent VH-IVUS of a non-culprit artery. The primary endpoint comprised a composite of ACS, unplanned revascularisation or death. Of 724 lesions identified by VH-IVUS, 271 (37 %) satisfied criteria for VH-TCFA. At 1-year follow-up, 56 patients had at least one event, 11 had a culprit lesion-related event (which were discounted), 27 had a definite non-culprit lesion-related event and the remaining 18 had an indeterminate event (an event that could not be judged to be either culprit or non-culprit lesion related). The presence of VH-TCFA was independently associated with the composite endpoint (adjusted HR 1.98, 95 % CI 1.09–3.60; *p* = 0.026), as was plaque burden (adjusted HR 2.90, 95 % CI 1.60–5.25; *p* < 0.001). In addition, the ATHEROREMO-IVUS was the first study to demonstrate that the presence of VH-TCFA in non-culprit vessels is associated with the hard endpoints of death and ACS at 1 year, not just unplanned rehospitalisation or revascularisation. This study also demonstrated that it may not be necessary to image all three coronary arteries to gain information about the pan-coronary vulnerability of the patient.

Together, these landmark studies demonstrate the prognostic potential of VH-IVUS; illustrating how information acquired using this imaging modality regarding plaque burden, minimal luminal area and the presence of TCFA, could be used to incrementally stratify risk in patients with proven CAD.

### IVUS and Calcified Plaques

Recently, Amano et al. performed a cross-sectional study to evaluate the association between angiographic plaque calcification and vulnerability via the identification of TCFA using VH-IVUS in *n* = 140 consecutive patients with ACS [41]. Patients were divided into four groups according to degree of calcification: (i) none (no calcium detected in any lesion; *n* = 37), spotty (circumferential calcium deposits <90°; *n* = 65), intermediate (calcium deposits ≥90° but <180°; *n* = 37) and extensive (calcium deposits ≥180°; *n* = 16). The mean number of VH-TCFA in the spotty calcification group was significantly greater than that in the no calcification, intermediate and extensive groups (0.66 ± 0.71 vs. 0.22 ± 0.42, *p* < 0.01; 0.32 ± 0.48, *p* < 0.05; and 0.13 ± 0.34, *p* < 0.01, respectively). In addition, the group with spotty calcification had a significantly greater necrotic core compared to that with angiographic calcification (*p* < 0.05). The presence of angiographic and VH-IVUS calcification correlated strongly (*p* < 0.05). These data suggest that calcified lesions are highly vulnerable; those with spotty or intermediate calcification on VH-IVUS, without angiographic calcification, appear to be more vulnerable than those with angiographic calcification. However, care should be taken with these results, due to the potential issues with the misclassification of necrotic core and dense calcium on the VH-IVUS algorithm.

## OCT

### OCT Technology

OCT, which uses the reflection of near-infrared light to generate an image, offers unparalleled spatial resolution (<10 μm axial; 20–40 μm lateral) and is a core intravascular imaging modality in clinical practice [42, 43]. OCT uses a low-coherence, near-infrared light source (centre wavelength, *λ*_o_ = 1.3 μm) which is directed at and reflected from the vessel wall. The complex principles underlying image generation have been previously described [44]. The OCT catheter contains an optical fibre, which rotates to acquire an image, and is mounted on an automated pullback device, which images the artery in a helical fashion (Fig. 1b). OCT enables high-resolution imaging at the cost of significant artefact due to blood cells, which scatter light and attenuate the image. Therefore, a bloodless field is required for optimal image acquisition.

Two main methods of OCT are in existence: first-generation time domain OCT (TD-OCT), which has largely been superseded by second-generation frequency domain OCT (FD-OCT) [43]. TD-OCT generates an image by sequentially measuring isolated near-infrared optical reflections at various depths, with the use of a moving reference mirror. However, this technique is slow (e.g. with pullback speed 1–5 mm s^−1^; image acquisition ≥45 s) and requires either proximal balloon occlusion (potentially pro-arrhythmogenic) or continuous flushing with iso-osmolar contrast to obtain artefact-free images. Frequency domain OCT uses a near-infrared source capable of generating *λ*_o_ = 1.25–1.35 μm at a single point, recording reflections at different depths without movement of the reference mirror. Depth profiles, reconstructed using Fourier transformation, permit 10-fold faster acquisition (e.g. with pullback speed 15–40 mm s^−1^; image acquisition 3–5 s), without proximal occlusion and minimising contrast injection.

OCT enables high-resolution characterisation of the vascular layers within a healthy artery and can identify morphological changes to the vessel surface associated with high-risk, vulnerable atherosclerotic plaques, including fibrous, lipid-rich and calcified lesions, in addition to red and white thrombus, and macrophages (Fig. 1b) [45, 46]. Potential disadvantages of OCT include a limited depth of penetration (1–2.5 mm) particularly through lipid-rich lesions, the inability to image the adventitia to assess plaque burden, the requirement for a blood-free field and common imaging artefacts [43, 44].

## OCT and Vulnerable Plaques

### Definitions

As the axial resolution of OCT is much greater than the diagnostic cut-off for the thin fibrous cap of TCFA, OCT is well placed to identify these in vivo. However, an ongoing debate exists with regard to the precise definition of a TCFA on OCT imaging: there is significant discrepancy between TCFA morphology on histological vs. OCT analysis [47]. In a study of *n* = 43 patients with ACS, Tanaka et al. utilised OCT to characterise plaque rupture at the culprit site. Using a cut-off of 70 μm, the authors demonstrated that 67 % of ruptured plaques demonstrated a thin fibrous cap, in stark comparison to the ≥95 % quoted in histopathological studies [6, 48]. It has been hypothesised that this may be a consequence of the shrinkage of pathological specimens; the true thickness of a TCFA cap may be greater in vivo. Although some studies advocate that the arc of the underlying lipid pool in TCFA should subtend an angle of ≥90°, there is no consensus that this should be incorporated into the definition of OCT-TCFA.

### OCT-TCFA in ACS

OCT has been utilised to accurately detect and discriminate between various plaque compositions, predicting peri-PCI complications [46]. A case-control study comparing 26 patients with ST-elevation MI to 16 with stable angina demonstrated that the ACS patients had greater incidence of OCT-TCFA in the culprit lesion (85 vs. 13 %, *p* < 0.001), with a reduced fibrous cap wall diameter (57 ± 12 vs. 180 ± 65 μm, *p* < 0.001) [49]. This was replicated in similar studies, comparing OCT-TCFA prevalence in ST-elevation vs. non-ST-elevation ACS (78 vs. 49 %, respectively; *p* = 0.008) [50] and in unstable vs. stable angina pectoris (81 vs. 47 %, respectively; *p* = 0.002) [51]. In each study, the average cap thickness was less for the group with greater disease severity, i.e. average thickness in STEMI < NSTEMI < unstable angina < stable angina, respectively [49–51].

In addition, OCT may be used to differentiate between TCFA and eroded or calcified plaques [45]. The features associated with plaque erosion, such as luminal thrombus with absent surface endothelium, have been correlated with sudden cardiac death [52, 53]. While OCT may correctly identify an irregular luminal surface, the resolution of OCT is inadequate to visualise endothelium [44, 53]. The use of OCT to detect plaque erosion may be particularly beneficial in ST-elevation MI to guide thrombectomy [54].

### OCT and Neoatherosclerosis

OCT has permitted new insights into the pathophysiology of neoatherosclerosis, in-stent restenosis and thrombosis. Initially thought to be associated with the early period after bare-metal stent deployment, evidence of in-stent restenosis has emerged both early and late following insertion of bare-metal and drug-eluting stents and can present as ACS [55, 56]. OCT imaging following stent deployment demonstrates the transformation of the neointima (which forms as a result of stent neovascularisation) to acquire features of lipid-rich, vulnerable plaque: a process termed neoatherosclerosis [57]. In one study, *n* = 50 patients with in-stent restenosis, and intimal hyperplasia >50 % of drug-eluting stent area, underwent OCT, which demonstrated the presence of TCFA in over half [57]. In a further study, serial OCT observed that approximately one third of patients with homogenous neointimal proliferation demonstrated progression to neoatherosclerosis [58]. As a result, patients with neoatherosclerosis are more likely to develop adverse sequelae, e.g. stent thrombosis and ACS, than those without [59, 60].

### OCT in Vulnerable Plaque Monitoring

The efficacy of pharmacotherapeutic agents in stabilising vulnerable plaques and preventing rupture has been evaluated using OCT. Takarada et al. used serial OCT imaging to compare plaque morphology in *n* = 40 patients, allocated to receive either statin therapy or no statin treatment, post-MI [61]. At 9 months, the authors report increases in fibrous cap depth in both groups, with a greater benefit observed in statin-treated patients (192 ± 41 vs. 25 ± 8 μm, respectively; *p* < 0.001). However, the significance of these changes with regard to clinical outcome is uncertain. A further, prospective trial, evaluating the effect of statin pharmacotherapy on vulnerable plaque morphology in *n* = 42 patients with stable angina demonstrated a significant increase in fibrous cap thickness in a statin-treated vs. dietary modification group (52 ± 32 vs. 2 ± 22 μm, respectively; *p* < 0.001) [62]. No differences were reported in clinical outcome at a median follow-up of 9 months; no patients experienced MI or cardiac-related death, and target vessel revascularisation was similar in both groups (15.4 vs. 18.7 %, respectively; *p* = 0.776). Nishio et al. randomised *n* = 30 patients with untreated dyslipidaemia and ≥1 TCFA on baseline OCT to receive statin therapy with or without eicosapentaenoic acid, observing that despite similar levels of low-density lipoprotein at follow-up, patients that received eicosapentaenoic acid experienced greater increases in cap thickness vs. controls (54.8 ± 27.9 vs. 23.5 ± 11.6 μm, respectively; *p* < 0.0001) [63]. However, while OCT can provide evidence of positive morphological improvements to TCFA, further, longitudinal investigations, with adequate sample sizes, are required to establish if these changes improve clinical outcomes.

## NIRS

### NIRS Technology

NIRS is a recently developed imaging modality, first tested in vivo in humans in 2001, and is developed for clinical applications over the past decade [64, 65]. NIRS uses a catheter-mounted core of optical fibres to emit and receive diffuse reflectance near-infrared light (*λ*_o_ = 0.8–2.5 μm) [66, 67]. Similar to OCT, the NIRS catheter contains an optical fibre which rotates (240 rotations min^−1^) to acquire an image and is mounted on an automated pullback device. The emitted wavelengths are absorbed in a specific pattern by each component of an atherosclerotic plaque. The diffuse reflectance signals from the tissue are converted to spectra, which undergo algorithmic transformation into a chemogram: a map of the arterial wall, which displays signals suggestive of lipid-core plaque (LCP) [68]. LCP is defined as a plaque ≥2 mm in length, with arterial circumference ≥60° [69]. The *x*-axis represents the position of the catheter relative to the start of the pullback; the *y*-axis indicates the degree of rotation [70, 71]. Red and yellow demonstrate low and high probability of LCP presence, respectively [72, 73]. The chemogram undergoes further processing to yield a summary, *block chemogram*, providing an interpretation of results for each 2-mm segment of an artery. The same colours are used for the chemogram, which are dependent on the probability of LCP in a given 2-mm region of an artery: red (*p* < 0.57), orange (0.57 ≤ *p* < 0.84), tan (0.84 ≤ *p* < 0.98) and yellow (*p* ≥ 0.98) [74]. Unlike the aforementioned imaging techniques, NIRS penetrates effectively through calcium and stents. It does not require post-processing nor a bloodless operating field. However, that NIRS provides only compositional insight (i.e. the probability of a lipid-core plaque at a given site) without any quantitative or morphological data (e.g. lumen size, plaque volume) is a limitation [75].

### Algorithm Derivation and Validation

The algorithm for lipid detection was calibrated using post-mortem coronary arteries [71]. Gardner et al. performed NIRS within 212 segments of a coronary artery (from 84 ex vivo hearts), followed by histopathological analysis of sections taken at 2-mm intervals. The first 33 hearts were used to develop the algorithm; the remaining 51 were used in prospective, double-blind fashion to evaluate NIRS in identifying LCP. The algorithm identified LCP with a receiver-operator characteristic area of 0.80 (95 % CI 0.76–0.85) and sensitivity and specificity of 49 and 90 %, respectively [71]. The SPECTACL (SPECTroscopic Assessment of Coronary Lipid) study was performed to validate the NIRS algorithm in vivo in *n* = 106 patients. This study demonstrated the feasibility and safety of spectral data collection via NIRS, with reasonable sensitivity and specificity [67].

## NIRS and Vulnerable Plaques

### LCP, NIRS and ACS

The ATHEROREMO-NIRS (European Collaborative Project on Inflammation and Vascular Wall Remodelling in Atherosclerosis-Near-Infrared Spectroscopy) study was performed to assess the prognostic value of NIRS [76••]. NIRS was performed in a non-culprit coronary artery in *n* = 203 patients undergoing PCI for stable angina or ACS; LCP within the vessel was measured as the maximal lipid-core burden index (LCBI). Patients were followed up for a median duration of 1 year for a composite endpoint of all-cause mortality, non-fatal ACS, stroke and unplanned coronary revascularisation. Patients with LCBI greater than the median value experienced a significantly greater rate of the composite endpoint vs. patients with LCBI less than the median value (16.7 vs. 4.0 %, respectively; adjusted HR 4.04, 95 % CI 1.33–12.29; *p* = 0.01).

The prospective, multi-centre COLOR (Chemometric Observation of Lipid-Core Plaques of Interest in Native Coronary Arteries Registry) registry was established to evaluate the association between the presence of LCP and peri-procedural MI [74]. Sixty-two patients with stable pre-procedural cardiac biomarkers underwent NIRS prior to PCI. Peri-procedural MI (defined as a cardiac biomarker rise greater than or equal to three times the upper limit of normal) occurred in 7 patients with large LCP (LCBI ≥500; *n* = 14), compared to 2 patients with lower LCP (*n* = 48) giving a relative risk of 12 (95 % CI 3.3–48; *p* = 0.0002), demonstrating a significant association between high LCP burden and peri-procedural MI.

The recent prospective, multi-centre CANARY (Coronary Assessment by Near-infrared of Atherosclerotic Rupture-prone Yellow) study demonstrated that treatment with PCI reduced LCP burden [77]. Patients experiencing peri-procedural MI had greater LCBI vs. patients without MI (481.5 vs. 371.5; *p* = 0.05). In addition, the effectiveness of a distal protection device in preventing peri-procedural MI was assessed in 31 lesions with LCBI ≥600. Patients were randomised to receive PCI with distal protection (*n* = 14) vs. PCI without distal protection (*n* = 17). The use of distal protection device had no effect on the rate of peri-procedural MI (*p* = 0.69).

## Comparing Established Imaging Techniques

Several studies have investigated the comparative accuracy of different imaging modalities to detect vulnerable coronary plaque. A recent study by Kini et al. compared IVUS, OCT and NIRS in *n* = 110 patients with established CAD, with regard to the prediction of peri-procedural MI [78•]. In patients that developed peri-procedural MI (*n* = 10), OCT-derived minimum fibrous cap thickness was significantly lower compared to those that did not develop MI (55 vs. 90 μm, respectively; *p* < 0.01). Both IVUS-measured plaque burden (84 ± 9 vs. 77 ± 8 %; *p* < 0.01) and NIRS-measured LCBI (556 vs. 339, *p* < 0.01) were greater in the peri-procedural MI group. The authors performed multivariate logistic regression, identifying OCT cap thickness as the only predictor of peri-procedural MI (odds ratio (OR) 0.91, 95 % CI 0.81–0.98; *p* = 0.04). When removed from the model, the IVUS- and NIRS-derived measures became significant predictors. Thus, OCT-based fibrous cap thickness, suggesting significant plaque vulnerability, is the most significant predictor of peri-procedural MI.

Numerous studies demonstrate that, while the absolute, in vivo diagnostic sensitivity of each imaging technique is favourable, the synergistic use of multiple imaging methodologies may be advantageous. Indeed, to this end, active preclinical and clinical research is ongoing to combine imaging modalities.

### Hybridisation of IVUS and OCT

Many investigators have speculated that the fusion of IVUS and OCT would prove an optimal imaging strategy. The enhanced resolution of OCT would enable the precise assessment of luminal morphology (i.e. identification of cap thickness, thrombus and plaque erosion), whereas the increased penetration of IVUS would allow the evaluation of plaque burden, architecture and remodelling. The combined usage of IVUS and OCT is proven to improve high-risk plaque detection in multiple studies. Sawada et al. used both VH-IVUS and OCT to detect TCFA in *n* = 56 patients with established angina. In total, 126 lesions were evaluated, demonstrating that each modality had an adequate absolute sensitivity and specificity for identifying TCFA, which improved when combined [79]. Similarly, Gonzalo et al. demonstrated improved diagnostic precision using VH-IVUS and OCT [80]. Although single-catheter IVUS-OCT systems are in development, significant barriers remain.

### Hybridisation of VH-IVUS and NIRS

Although enabling characterisation of plaque architecture, IVUS has limited capability in identifying plaque composition. VH-IVUS analysis provides more data but is subject to underestimation of lipid tissue behind calcified plaques and in stent segments. Brugaletta et al. performed a study comparing VH-IVUS and NIRS in *n* = 31 patients with angina, demonstrating poor concordance between the identification of NC on VH-IVUS and LCP on NIRS, despite good sensitivity and specificity for the individual techniques, and the prior association of both entities with adverse events [73]. A study by Pu et al. observed similar findings [81]. Thus, the fusion of these technologies may improve detection of vulnerable plaque. The integration of an ultrasound image to demonstrate morphology, with a chemogram to identify lipid-rich regions, is theoretically advantageous, although evidence is limited regarding the incremental diagnostic utility of this technique. It is currently being used to assess statin-mediated plaque regression in IBIS-3 (Integrated Biomarker and Imaging Study-3) [82].

## Other Imaging Techniques

In addition to established imaging modalities, there are many approaches in active, preclinical and clinical development. We briefly review some of the more prominent technologies.

### Coronary Angioscopy

Using high-resolution (10–50-μm) fibre optics, coronary angioscopy permits the visualisation of the arterial intima and plaque surface [83]. Although capable of assessing superficial characteristics (such as colour), identifying red and white thrombus and detecting localised damage to the vessel wall, (e.g. flaps, fissure, ulceration), angioscopy provides limited insight into plaque morphology. Preliminary data suggests that plaque colour, related to fibrous cap thickness, may be associated with vulnerability (intensely yellow plaques have thinner fibrous caps, predisposing to rupture) [84]. However, angioscopy requires a bloodless field and is highly operator dependent, limiting routine application. As such, coronary angioscopy is primarily limited to research use.

### Intravascular MRI

The development and application of an intravascular MRI probe, integrated within the tip of a coronary catheter and capable of identifying TCFA in intact, post-mortem coronary arteries, was demonstrated by Schneiderman et al. [85]. This proof-of-concept device contained both a magnet and coil within a 1.73 mm diameter. The probe produced axial images, with penetration of 250 μm, and achieved an estimated resolution of 100 μm. In preliminary analysis of post-mortem vessels, intravascular MRI identified vulnerable lesions with a sensitivity and specificity of 100 and 89 %, respectively. However, long image acquisition times (2.5–4 min), requiring occlusion of the artery, have proven a barrier to translating this technology into in vivo applications.

### Raman Spectroscopy

Raman spectroscopy identifies organic molecules, using the Raman shift principle, the inelastic scattering of monochromatic light from a laser source, when it reflects off a substance. The unique molecular characteristics of each plaque constituent, particularly lipid and calcium, and their corresponding Raman shift patterns, enable Raman spectroscopy to sensitively detect a coronary plaque [86]. An algorithm, derived from the relative contributions of the independent spectra of the constituents of an atherosclerotic plaque, has been validated [87]. However, intracoronary applications have numerous limitations, such as the small proportion of photons that become shifted, resulting in a poor signal-to-noise ratio and limited tissue penetration.

### Near-Infrared Fluorescence Molecular Imaging

Using technology similar to NIRS, intravascular near-infrared fluorescence molecular imaging utilises the phenomenon of molecular fluorescence to identify specific regions of arterial inflammation and injury [88]. Near-infrared fluorescence (NIRF) permits a targeted approach to the identification of plaque components (e.g. lipid core). Preclinical, proof-of-concept studies have used NIRF catheters, in conjunction with a cysteine protease imaging reporter, for in vivo intracoronary imaging in animal models. Jaffer et al. report high-resolution spatial mapping of arterial inflammation, superimposed on IVUS imaging, to identify high-risk plaque regions [89].

A comparison of all imaging technologies described in this review is presented in Table 1.

**Table 1:** Comparison of imaging technologies for the detection of vulnerable coronary plaques

Imaging technique	Imaging technology	Wavelength (μm)	Penetration (mm)	Resolution (μm)	Clinical utility in detection of vulnerable plaque feature				
					Fibrous cap	Lipid core	Inflammation	Calcium	Thrombus
Coronary Angiography^a^	X-ray	0.00001–0.01	0.0	>500	−	−	−	+	+++
(IB/VH-)IVUS^a^	Ultrasound	35–80	10.0	100–200	+	++	−	+++	−
OCT^a^	Infrared	1.3	1.0–2.5	<10	+++	+++	+	++	++
NIRS	Near-infrared	0.8–2.5	1.0–2.0	N/A	−	+++	−	−	−
Raman spectroscopy	Near-infrared	0.75–1.0	1.0–2.0	N/A	−	+++	−	−	−
IV-MRI	MRI	N/A	0.25	100	+	++	++	++	+
Angioscopy	Optical	0.4–0.7	0.0	10–50	+	+	−	−	+++
Thermography	Infrared	0.8–2.5	1.0	500	−	−	+++	−	−

− = not possible; + = adequate; ++ = good; +++ = excellent

*IB* integrated backscatter, *IV-MRI* intravascular MRI, *IVUS* intravascular ultrasound, *mm* millimetres, *N/A* not applicable, *NIRS* near-infrared spectroscopy, *OCT* optical coherence tomography, *μm* micrometres

^a^In routine clinical use

## Clinical Guidelines

Evidence-based clinical practice guidelines, regarding the usage of intracoronary imaging, have been developed by the ACC/AHA/SCAI and ESC/EACTS. The 2011 North American guidelines recommend the use of IVUS which is reasonable for the assessment of (i) angiographically indeterminate left main CAD (class IIa, level of evidence B) and (ii) non-left main coronary arteries with angiographically intermediate stenoses (50–70 %; class IIb, level of evidence B) [90]. The guidelines suggest other situations in which the IVUS may have clinical utility, such as in the evaluation of the aetiology of stent restenosis (class IIa, level of evidence C) and thrombosis (class IIb, level of evidence C). The routine use of IVUS for lesion evaluation, when PCI is otherwise not intended, is not recommended (class III). Although the guideline acknowledges OCT, it does not provide recommendations for usage outside of clinical research. No recommendations are made regarding other imaging modalities. The 2014 European guidelines advocate IVUS in selected patients (i) to optimise stent placement (class IIa, level of evidence B) and (ii) to assess severity and optimise treatment of unprotected left main lesions (class IIa, level of evidence B) [91]. The guideline also advises consideration of IVUS and/or OCT to detect stent-related mechanical problems (e.g. restenosis, thrombosis; class IIa, level of evidence C) and OCT in selected patients to optimise stent implantation (class IIb, level of evidence C). No recommendations are made regarding other imaging modalities. Both recommendations advocate the need for further clinical research to demonstrate that the use of intracoronary imaging techniques improves clinical outcomes.

## Conclusion

The development of invasive intracoronary imaging, exemplified by IVUS, OCT and NIRS, has revolutionised an understanding of the pathophysiology of coronary artery disease. Translation into the cardiac catheterisation lab has permitted detailed, in vivo identification and evaluation of high-risk, vulnerable coronary plaques in both clinical research and cardiology practice. Of the available array of imaging modalities, each has distinct advantages and disadvantages: VH-IVUS enables precise characterisation of plaque architecture but lacks spatial resolution. OCT permits high-resolution measurement of the fibrous cap but has limited penetration through the arterial wall. NIRS robustly detects lipid cores but provides limited quantitative data regarding the coronary lumen or plaque morphology. Hybrid approaches negate these limitations and are gaining traction in early clinical studies. Numerous novel imaging technologies are under development. However, while improved image quality is imperative, nascent techniques must add value to existing approaches. Robust clinical trials are required to evaluate whether invasive intracoronary imaging improves patient outcomes.

## Abbreviations

ACS: Acute coronary syndrome
CAD: Coronary artery disease
DC: Dense calcium
FFT: Fibro-fatty tissue
FT: Fibrous tissue
IB-IVUS: Integrated backscatter intravascular ultrasound
IVUS: Intravascular ultrasound
LCBI: Lipid-core burden index
LCP: Lipid-core plaque
MI: Myocardial infarction
NC: Necrotic core
NIRS: Near-infrared spectroscopy
OCT: Optical coherence tomography
PCI: Percutaneous coronary intervention
TCFA: Thin-cap fibroatheroma
VH-IVUS: Virtual histology intravascular ultrasound
